# Supplementary material for: Prospective Measures of Adherence by Questionnaire, Low Immunosuppression and Graft Outcome in Kidney Transplantation
Source: J Clin Med. 2021 May 10;10(9):2032. doi: 10.3390/jcm10092032 (PMC8125965; doi:10.3390/jcm10092032)
Supplement: Supplementary file 1 [file jcm-10-02032-s001.zip › jcm-1174822-supplementary.pdf]

Table S1. Association between CNI trough level and treatment adherence assessed by the Morisky scale

	Non adherent patients*	Adherent patients*	p-value‡
**Treatment by tacrolimus**			
At 3 months	8.7 (2.0)	9.2 (2.7)	0.33
At 6 months	8.4 (2.0)	8.2 (2.1)	0.58
At 12 months	7.6 (2.4)	7.9 (2.6)	0.55
At 24 months	7.9 (2.5)	7.6 (2.4)	0.44
**Treatment by cyclosporine**			
At 3 months	147.3 (30.0)	164 (46.0)	0.31
At 6 months	147.1 (42.3)	147.3 (52.6)	0.99
At 12 months	123.1 (41.9)	127.3 (45.6)	0.77
At 24 months	87.8 (34.2)	98.4 (46.4)	0.52

*assessed by the Morisky scale at the time of CNI trough levels measures
‡assessed by Student test

Table S2. Factors associated with occurrence of acute rejection over the 5 years post transplantation.

	N	No rejection (n=228)	At least one rejection (n=73)	P value
**Age** (years), mean (SD)	301	50.0 (12.3)	47.8 (15.4)	0.27
**Male,** n (%)	301	154 (67.5)	54 (74.0)	0.30
**Primary kidney disease**	295			0.42
Vascular, n (%)		8 (3.6)	2 (2.8)	
Diabectics, n (%)		32 (9.4)	3 (4.2)	
Glomerulonephritis, n (%)		62 (27.8)	20 (27.8)	
Tubulo-interstitial, n (%)		24 (10.8)	7 (9.7)	
Hereditary (including polykystic kidney disease), n (%)		51 (22.9)	13 (18.1)	
Other or undetermined, n (%)		57 (25.6)	27 (37.5)	
**Number of previous graft(s)**	271			0.71
0, n (%)		167 (81.9)	57 (85.1)	
1, n (%)		31 (15.2)	10 (14.9)	
≥ 2, n (%)		6 (2.9)	0 (0.0)	
**Sensitized patients**, n (%)	226	45 (27.1)	16 (26.7)	0.95
**Donor age** (years), mean (SD)	278	49.0 (15.6)	49.7 (16.8)	0.76
**Living donor,** n (%)	298	8 (3.5)	1 (1.4)	0.69
**Expanded criteria donor,** n (%)	274	86 (41.8)	30 (44.1)	0.73
**Number of HLA mismatches (A-B-DR)**	295			0.22
0 - 1, n (%)		11 (4.9)	2 (2.9)	
2 - 4, n (%)		131 (58.2)	34 (48.6)	
5 - 6, n (%)		83 (36.9)	34 (48.6)	
**Total ischemia time** (hours), mean (SD)	269	16.2 (6.4)	16.5 (5.7)	0.72
**Induction treatment**	276			0.60
Basiliximab, n (%)		126 (60.0)	42 (63.6)	
rATG, n (%)		84 (40.0)	24 (36.4)	
**Delayed graft function,** n (%)	292	46 (20.6)	15 (21.7)	0.84
**Maintenance treatment at 3 months post-transplant**	271			**0.002**
Cyclosporine		42 (18.6)	18 (40.0)	
Tacrolimus		181 (80.1)	25 (55.6)	
Without CNI		3 (1.3)	2 (4.4)	
**Maintenance treatment at 6 months post-transplant**	255			**0.01**
Cyclosporine		45 (20.1)	10 (32.3)	
Tacrolimus		175 (78.1))	18 (58.1)	
Without CNI		4 (1.8)	3 (9.7)	
**Maintenance treatment at 12 months post-transplant**	241			**0.005**
Cyclosporine		42 (19.0)	9 (45.0)	
Tacrolimus		171 (77.4)	9 (45.0)	
Without CNI		8 (3.6)	2 (10.0)	
**Maintenance treatment at 24 months post-transplant**	227			0.39
Cyclosporine		41 (19.0))	3 (27.3)	
Tacrolimus		164 (75.9)	7 (63.6)	
Without CNI		11 (5.1)	1 (9.1)	
**Corticosteroids at 3 months**	301			0.06
Continued		188 (82.5)	67 (91.8)	
Discontinued		40 (17.5)	6 (8.2)	

N : subjects with available data ; SD : standard deviation ; HLA : Human Leucocyte Antigen

Table S3. Effect of non-adherence measured by the Morisky scale and immunosuppression regimen on the hazard of acute rejection. Results of Cox proportional hazard model. Multivariable analysis.

	3 months (N = 246)			6 months (N = 228)			12 months (N = 220)			24 months (N = 189)		
	HR*	CI 95%	p-value	HR*	CI 95%	p-value	HR*	CI 95%	p-value	HR*	CI 95%	p-value
**Non adherent *vs* adherent**†	0.93	0.40-2.15	0.94	1.19	0.52-2.75	0.77	0.52	0.17-1.59	0.27	0.57	0.11-2.91	0.60
Maintenance treatment			0.001			0.0008			0.005			0.52
Tacrolimus	1			1			1			1		
Cyclosporine	**2.68**	**1.42-5.08**		2.08	0.92-4.71		**4.07**	**1.57-10.57**		1.60	0.31-8.27	
Without anticalcineurin	**4.48**	**1.27-17.7**		**6.97**	**1.99-24.41**		**5.78**	**1.21-27.5**		2.99	0.34-26.4	

HR: Hazard ratio ; CI: Confidence Interval ; N = Subjects with complete data, with a functioning graft at time of measure of NA and without acute rejection before this time ; †Subjects with at least one positive response to Morisky scale at time of measure. * adjusted for adherence status and maintenance treatment measured at the time mentioned above

Table S4. Factors associated with risk of graft failure defined as death, return to dialysis or retransplantation. Results of univariate Cox model.

	N	HR	CI 95%	p-value
**Recipient age, per year**	297	1.02	0.99-1.03	0.08
**Female sex**	297	0.71	0.43-1.16	0.17
**Primary kidney disease**	292			0.98
Vascular, n (%)		1		
Diabetics, n (%)		1.10	0.28-4.25	
Glomerulonephritis, n (%)		1.10	0.33-3.65	
Tubulo-interstitial, n (%)		0.87	0.23-3.28	
Hereditary (including PKD), n (%)		0.81	0.24-2.79	
Other, n (%)		0.91	0.26-3.22	
Undetermined, n (%)		1.14	0.32-4.15	
Multiples, n (%)		0.76	0.08-7.25	
**Number of previous grafts, per unit**	269	0.93	0.57-1.51	0.77
**Preemptive transplantation**	296	0.59	0.24-1.47	0.26
**Transplant with a living donor**	295	0.33	0.05-2.41	0.28
**Donor age, per year**	275	1.02	1.01-1.04	**0.007**
**Transplant with an expanded criteria donor**	271	1.48	0.95-2.30	0.08
**Number of HLA mismatches, per unit**	292	1.00	0.85-1.18	0.968
**Induction treatment**	272			0.47
Basiliximab, n (%)		1		
rATG, n (%)		1.19	0.74-1.91	
**Total ischemia time**, per hour	266	1.02	0.98-1.05	0.38
**Occurrence of a delayed graft function**	289	1.38	0.83-2.29	0.21
**Maintenance treatment at 3 months**	296			0.08
Tacrolimus		1		
Cyclosporine		**1.68**	**1.06-2.66**	
Without CNI		0.73	0.10-5.26	
**Maintenance treatment at 6 months**	294			0.10
Tacrolimus		1		
Cyclosporine		**1.62**	**1.01-2.60**	
Without CNI		0.50	0.07-3.62	
**Maintenance treatment at 12 months**	291			0.24
Tacrolimus		1		
Cyclosporine		1.51	0.91-2.51	
Without CNI		1.53	0.55-4.22	
**Maintenance treatment at 24 months**	282			0.23
Tacrolimus		1		
Cyclosporine		1.51	0.88-2.59	
Without CNI		1.64	0.65-4.12	
**CNI trough levels at 3 months**	239			0.93
Standard CNI through levels		1		
Low CNI through levels		1.09	0.34-3.46	
Without CNI		0.71	0.10-5.13	
**CNI trough levels at 6 months**	253			0.66
Standard CNI through levels		1		
Low CNI through levels		1.22	0.56-2.67	
Without CNI		0.48	0.07-3.44	
**CNI trough levels at 12 months**	253			0.25
Standard CNI through levels		1		
Low CNI through levels		1.62	0.84-3.12	
Without CNI		1.66	0.60-4.60	
**CNI trough levels at 24 months**	248			**0.007**
Standard CNI through levels		1		
Low CNI through levels		**2.36**	**1.37-4.07**	
Without CNI		1.84	0.73-4.67	
**Corticosteroids discontinued at 3 months post transplant**	296	0.99	0.55-1.78	0.96

**Occurrence of *de novo* DSA between 2 and 3 years post transplantation**	219	3.26	1.70-6.28	**0.0004**
**Occurrence of acute rejection**	297	2.10	1.35-3.26	**0.001**

N : number of available data ; HR : Hazard Ratio ;

Table S5. Effect of non-adherence measured by the Morisky scale and immunosuppression regimen (standard CNI trough level / low CNI trough level / any anticalcineurin) on the hazard of graft failure since the third year post transplantation. Results of Cox proportional hazard model. Multivariable analysis.

	**3 months (N‡ = 155)**			**6 months (N‡ = 158)**			**12 months (N‡ = 168)**			**24 months (N‡ = 149)**		
	HR*	CI 95%	p-value	HR*	CI 95%	p-value	HR*	CI 95%	p-value	HR*	CI 95%	p-value
**Non adherent *vs* adherent†**	1.03	0.40 – 2.62	0.98	1.01	0.45 – 2.24	0.85	0.77	0.37 – 1.62	0.45	0.97	0.49 – 1.92	0.99
Recipient age (*per* year)	0.99	0.96 – 1.04	0.75	1.01	0.97 – 1.05	0.68	1.02	0.98 – 1.05	0.42	0.99	0.96 – 1.03	0.79
Donor age			0.54			0.77			0.88			0.25
	1			1			1			1		
	1.53	0.50 – 4.62		1.23	0.41 – 3.66		1.19	0.42 – 3.34		2.11	0.62 – 7.17	
	2.43	0.72 – 8.15		1.75	0.58 – 5.31		1.52	0.50 – 4.66		2.87	0.80 – 10.29	
	2.70	0.63 – 11.61		1.32	0.34 – 5.18		1.63	0.43 – 6.08		4.41	1.05 – 18.57	
**Maintenance treatment**			0.42			0.29			0.78			0.20
Standard CNI trough level ©	1			1			1			1		
Low CNI trough level ®	0.48	0.10 – 2.40		1.17	0.39 – 3.50		0.87	0.39 – 1.96		1.88	0.94 – 3.76	
Without anticalcineurin	0.55	0.07 – 4.50		0.26	0.03 – 1.98		0.84	0.25 – 2.86		1.98	0.71 – 5.51	
Occurrence of acute rejection (time-dependent variable)	**2.13**	**1.04 – 4.35**	**0.03**	**2.25**	**1.14 – 4.44**	**0.01**	**1.93**	**1.01 – 3.68**	**0.04**	1.90	0.98 – 3.67	0.08
Occurrence of *dn*DSA between 2 and 3 years post-transplant	**2.66**	**1.09 – 6.45**	0.03	**3.74**	**1.55 – 9.04**	**0.001**	**2.39**	**1.07 – 5.37**	0.03	1.84	0.74 – 4.53	0.17

HR: Hazard ratio ; CI: Confidence Interval ; ‡Subjects with complete data and with a functioning graft at 3 years post-transplant; †Subjects with at least one positive response to Morisky scale at time of measure. © defined as tacrolimus trough levels ≥ 5 ng/ml or ciclosporin trough levels ≥ 100 ng/ml. ® defined as tacrolimus trough levels ≤ 5 ng/ml or ciclosporin trough levels ≤ 100 ng/ml. *adjusted for recipient and donor age, maintenance treatment at time of measure of NA, occurrence of acute rejection (time dependent variable) and occurrence of dnDSA between 2 and 3 years post transplant
